# Thermally Sprayed Coatings: Novel Surface Engineering Strategy Towards Icephobic Solutions

**DOI:** 10.3390/ma13061434

**Published:** 2020-03-21

**Authors:** Heli Koivuluoto, Enni Hartikainen, Henna Niemelä-Anttonen

**Affiliations:** Materials Science and Environmental Engineering, Faculty of Engineering and Natural Sciences, Tampere University, 33720 Tampere, Finland

**Keywords:** thermal spraying, polymer coatings, flame spraying, icephobicity, ice adhesion, wettability, coating design

## Abstract

Surface engineering promotes possibilities to develop sustainable solutions to icing challenges. Durable icephobic solutions are under high interest because the functionality of many surfaces can be limited both over time and in icing conditions. To solve this, one potential approach is to use thermally sprayed polymer or composite coatings with multifunctional properties as a novel surface design method. In thermal spraying, coating materials and structures can be tailored in order to achieve different surface properties, e.g., wetting performance, roughness and protection against several weathering and wearing conditions. These, in turn, are beneficial for excellent icephobic performance and surface durability. The icephobicity of several different surfaces are tested in our icing wind tunnel (IWiT). Here, mixed-glaze ice is accreted from supercooled water droplets and the ice adhesion is measured using a centrifugal adhesion tester (CAT). The present study focuses on the icephobicity of thermally sprayed coatings. In addition, surface-related properties are evaluated in order to illustrate the correlation between the icephobic performance and the surface properties of differently tailored thermally sprayed coatings as well as compared those to other coatings and surfaces.

## 1. Introduction

Thermal spraying is used in various application fields for the production of protective coatings. ln this technology, almost all materials, e.g., metals, metal alloys, ceramics, hard metals, composites and polymers, can be used as a coating material as well as a substrate or base materials. Thermal spraying consists of different spray techniques such as flame, arc, high-velocity flame, plasma and cold gas dynamic spray processes [1]. The basic idea is the same in these different processes: coating material is melted or accelerated, sprayed on the surface, solidifying or deforming, and this way building-up a coating. Thermal spray processes can use thermal (combustion or electric arc) or kinetic energy (high velocity) or a combination of these for the coating formation [1,2]. Furthermore, this is a fast and robust coating manufacturing technology and suitable for many uses. For example, one interesting application field is to produce polymer coatings by thermal spraying. This way, solvent-based coating methods can be avoided in polymer coating production, acting as an environmentally friendly coating processing method. Polymer coatings are used, e.g., to increase corrosion, wear, and environmental resistance, and as slippery surfaces for reduction of friction [3,4,5]. They have shown great potential to have good icephobic properties, i.e., low ice adhesion, suitable wettability and freeze-thaw performance, which makes it easier to remove accreted ice on the surface. This is under high interest because surface engineering including thermal spraying could provide a sustainable approach for icing issues. In addition, the durability of current anti-icing solutions against environmental stresses and performance in all icing conditions is insufficient and thus, novel solutions are welcome.

Thermal spray coating solutions are under development to tackle the icing issues. These inflict serious problems for various industrial operations such as offshore industry, transport and cargo, ship industry, renewable energy production and aviation [6,7,8,9,10,11]. Ice accretion on the surfaces significantly reduces the efficiency, the safety and the operational tempo of different industrial processes. These detrimental icing events take place worldwide, e.g., in Scandinavia, Europe, Russia, Northern America, Japan and China [10,12]. The on-going climate change has also decreased sea ice coverage in the Arctic Ocean, which has considerably grown the industrial activity in this area [7]. The most typical examples of icing problems can be associated to the icing of superstructures of sea vessels and offshore platforms [6], ice accretion on wind turbines blades [13] and airplane wings [9] as well as ice loads on power network structures [10] and tall structures [11]. At the worst, icing is causing disasters, which have deep socioeconomic impacts, being hazardous not only for the environment but also for personnel.

It is important to find reliable solutions for these icing challenges. Active de-icing methods have been used to remove the ice, e.g., by using heaters, which can be produced with thermal spraying of metallic materials [14,15]. We have focused on passive anti-icing methods such as icephobic surfaces. The main idea of icephobic surfaces is to reduce the ice adhesion on the surface and prevent ice accumulation on the surface [16,17]. Surface engineering and coating technologies have shown potential results, but still more development is needed especially for durable coatings and surfaces, which are not losing their icephobicity under other environmental stresses such as rain, UV light, sand, other impurities or temperature exchanges. Many superhydrophobic surfaces have good icephobicity [18] but they might not be as resistant and durable as needed in environmental conditions. On the other hand, one of the latest icephobic surfaces, which have been under high research and interest, are slippery liquid impregnated porous surfaces (SLIPS) [19,20]. They have shown low ice adhesion values, acting as icephobic surfaces. However, if the porous solid layer is very thin, durability might be limited. Therefore, other surface engineering solutions are needed. Coating design can be varied in thermal spraying. Dense or porous coatings can be produced, depending on the requirements and needs of the surface and structure. In this study, we are producing smooth flame-sprayed polymer coatings, which have low ice adhesion as well as surfaces with even lower ice adhesion by combining thermal spraying and SLIPS strategy. Porous polymer coatings can be produced by using flame spraying and then, impregnated with the lubricant. Both coating design strategies have shown their suitability for icephobic purposes [21,22,23].

Generally, polymer materials have high chemical and environmental resistance [24]. Especially hydrophobic polymers have also potential to act as icephobic and slippery surfaces because they possess minimal water interaction and absorption. Extending this to the coatings, smooth and dense polymer coatings can be produced by thermal spraying [3,21,22]. Actually, flame spraying is one of the common thermal spray methods for spraying polymer coatings [3,5]. Process parameters especially temperature influence on coating formation. Donadei et al. [22] have noticed that lower process temperature by using high transverse speed and higher working distance will cause less polymer degradation during spraying, which is beneficial for icephobic behavior of the coatings. The advantages of thermally sprayed polymer coatings are related to the low cost and high performance of the coatings [3,25]. Flame-sprayed polyethylene (PE) coatings have previously been studied by Vuoristo et al. [26], where the research focused on the use of flame-sprayed PE coatings as natural gas pipeline coatings. On the other hand, ultra-high molecular weight polyethylene (UHMWPE) is known to have good protective properties and flame-sprayed UHMWPE coatings have also been studied [27]. Flame-sprayed PE coatings have primary applications in corrosion protection of components and metal structures as the alternatives for paints and metallic coatings. One benefit is also their applicability in difficult processing conditions [5].

In this study, we produce polymer coating by using thermal spraying and evaluate them based on their icephobic performance. Investigations are focusing on the icephobicity and wettability of thermally sprayed polymeric coatings and several reference materials and surfaces. In the icing tests, ice is accreted in an icing wind tunnel (IWiT) and ice adhesion measured with a centrifugal ice adhesion tester (CAT). One interesting focus point was the durable icephobic slippery liquid infused porous surfaces (SLIPS) designed and manufactured by using flame spraying (FS), utilizing polymeric materials, and further, impregnated with oil. These surfaces had low ice adhesions, which express their high icephobicity.

## 2. Materials and Methods 

### 2.1. Materials

Thermally sprayed icephobic surfaces were produced by using the flame spray (FS) process. Dense FS coatings were sprayed with relatively low gas flow rates in order to prevent overheating and burning of the powder with the flame. After spraying, a few post-heating passes with the flame were done without feeding powder to densify the structure and smoothen the surface. Commercially available and thermally sprayable polyethylene (PE, melting point 128 °C), PE mixed with fluoropolymer perfluoroethylene propylene (FEP, melting point 260–290 °C), ultra-high molecular weight polyethylene (UHMWPE, melting point 136 °C), polyether ether ketone (PEEK, melting point 343 °C) and polypropylene (PP, 160 °C) powders were used in these experiments. More information about flame spraying of PE, PE + FEB and UHMWPE coatings can be found from our previous study [21]. Furthermore, slippery liquid infused porous surfaces (SLIPS), combining porous FS PE coating with a lubricant was studied [23]. All FS coatings were produced using an oxygen–acetylene flame spray gun (Castodyn DS 8000, Castolin Eutectic, Lausanne, Switzerland). Gas pressure was 400 kPa for oxygen and 70 kPa for acetylene. Powder feeder (4MP, Oerlikon Metco, Pfäffikon, Switzerland) was used with compressed air as the carrier gas. Gas flows and spray distances were varied low to very low in order to achieve a porous FS PE structure. Rough PE porous surface was achieved with as-sprayed conditions whereas post-heating was done for smooth porous PE structure. After coating production, silicone oil (50 cSt, Sigma-Aldrich, Merck KGaA, Darmstadt, Germany) was impregnated into the structure in order to form the FS-SLIPS.

Other materials and coatings were studied for comparison. Bulk metals were mirror-polished aluminum (YH75, Hakudo Co., Tokyo Japan) and stainless steel (EN 1.4301/2B). Commercial paints tested included BladeRep (Alexit®, Mankiewicz Gebr. & Co., Hamburg, Germany), wind turbine paint (Carboline Ltd., St. Louis, MS, USA), Nanomyte® (NEI Corporation, Somerset, NJ, USA) and NeverWet® (NeverWet LLC, Lancaster, PA, USA). UltraEverDry® film (NetDesign s.r.o, Liberec, The Czech Republic) was representing a superhydrophobic surface. Bulk polymers were polyethylene (PEHWU, Simona AG, Kirn, Germany), polypropylene (PP-DWU AlphaPlus, Simona AG, Kirn, Germany), polytetrafluoroethylene (PTFE G400, Guarniflon, Castelli Calepio, Italy) and ultra-high molecular weight polyethylene (UHMW-PE, Tivar® 1000, Quadrant Group, Zurich, Switzerland). Other references were PTFE tape (PTFE Extruded Film Tape, 5490, 3M™, St. Paul, MN, USA), low-density polyethylene sheet (LDPE, thick film used for paper making [28]) and SLIPS containing thin polymeric membrane and infused silicone oil. PTFE and PP membranes (0.2 µm pore size, Sterlitech Inc., Kent, WA, USA) were used together with silicone oil (50 cSt, Sigma-Aldrich, Merck KGaA, Darmstadt, Germany). Preparation of these SLIPS surfaces is shown in our earlier research [20]. All coatings, materials and surfaces studied are summarized in Table 1.

### 2.2. Test Methods

Structures of FS coatings were analyzed with an optical microscope (Leica DM2500 M, Wetzlar, Germany) from the cross-sectional coating samples. Wetting behavior and water contact angle measurements were done with a drop shape analyzer (DSA100, Krüss, Hamburg, Germany). Static contact angle (CA), advancing contact angle (ACA) and receding contact angle (RCA), as well as contact angle hysteresis (CAH), were studied. The experiments were achieved by dispersing 5 µL water droplets of ultra-high purity water (MilliQ, Merck KGaA, Darmstadt, Germany) onto surfaces. In addition, roll-off/sliding angles were performed for SLIPS by tilting the surfaces with a 10 µm water droplet. The roughness of the solid and dry surfaces was analyzed by an optical profilometry (Alicona Infinite Focus G5, AT, Graz, Austria) using a 20× objective magnification, achieving a vertical resolution of 50 nm. The area of the measurements was 0.81 mm × 0.81 mm in the *XY*-plane and the results are presented as surface roughness, Sa, values.

**Figure 1:**
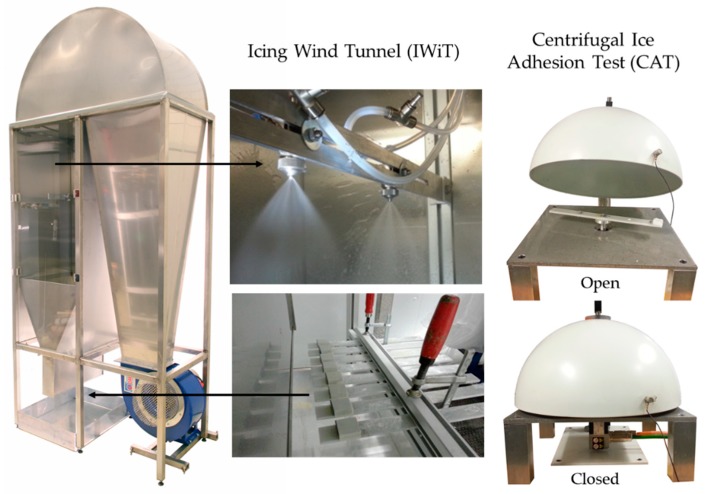
Icing Wind Tunnel (IWiT) and Centrifugal Ice Adhesion Test (CAT) at Tampere University.

Icing tests were done at Tampere University (TAU). The mixed-glaze ice type was accreted in the icing wind tunnel (IWiT) and the ice adhesion strength was examined with a centrifugal ice adhesion tester (CAT) [29]. Icing test systems are presented in Figure 1.

**Figure 2:**
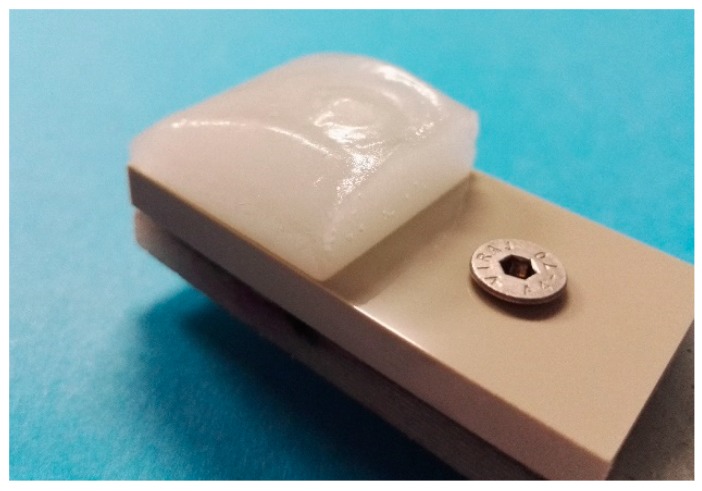
Mixed-glaze ice accreted in IWiT for CAT testing.

These test facilities are located in the cold climate room, where temperature can be set from room temperature to as low as −40 °C. All tested samples were 60 mm × 30 mm in size and the ice accreted area was 30 mm × 30 mm. Laboratory grade II+ water (Purelab Option-R 7/15, Elga, UK) was used for accreting the ice. The samples were let to cool down in the cold room prior to the ice accretion process. The most important parameters for the ice accumulation procedure for mixed-glaze ice used in this study were the ambient temperature of the cold room (−10 °C), the wind speed (25 m/s), and the supercooled water droplet size (~30 µm, as given by the nozzle manufacturer). In this study, the maximum water flow rate was 0.3 L/min. The accreted glaze ice refers to ice that has characteristics from both rime and glaze ice types, having more glaze-like features without icicles or runback ice. The mixed-glaze ice type forms a clean-cut rectangular structure. An example of the accreted ice is presented in Figure 2. In addition to this mixed-glaze ice, rime and glaze ice can be accreted in the IWiT as shown in our previous studies [21,29]. Ice adhesion strengths were determined using a CAT [29]. In this test, the centrifugal force detaches the ice from the surface and the ice adhesion strength (T) is calculated according to Equation (1):

*T = F/A = mr(αt)2/A* (1)

where a piece of ice of known mass *m* and contact area *A* is spun along a radial length *r*, which spins with a constant angular acceleration *α* of 300 rpm/s. From this equation, the ice adhesion strength (*T*) via shear stress is calculated at the time of detachment *t*.

## 3. Results and Discussion

**Figure 3:**
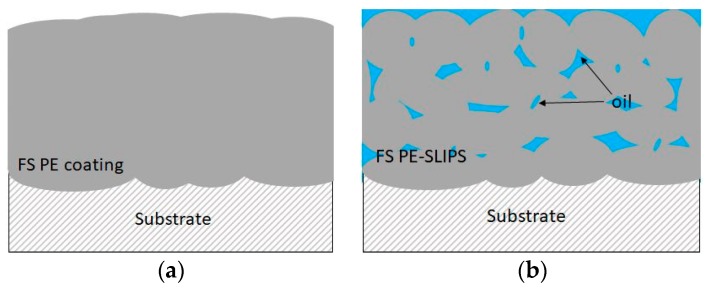
Schematic presentation of icephobic surfaces produced by using flame spraying (**a**) dense flame-sprayed (FS) polyethylene (PE) coating and (**b**) FS PE slippery liquid impregnated porous surfaces (SLIPS; porous structure with impregnated oil).

Thermal spraying is a versatile coating production technique. Traditionally, it is used, e.g., for corrosion and wear protection, thermal conductivity and insulation [3,30,31,32,33]. Lately, thermally sprayed coatings are developed for other advanced purposes, e.g., for self-cleaning and anti-icing [21,22,32]. Coatings and surfaces with good icephobic properties are potential to use in anti-icing applications, e.g., in wind energy, transportation, aviation and building industries. In our previous studies, flame spraying was used to produce polymer coatings with icephobic properties [20,21]. There are two approaches to achieve these goals. The first coating design approach is to produce dense and smooth polymer coatings and the second is to produce polymer coatings with porous structures and add lubricants to the structures, having slippery properties. The latter acts as SLIPS. Figure 3 shows a schematic presentation of these two flame spray approaches towards anti-icing solutions. We are focusing on thermal spraying because durable coatings with a high variety of coating thicknesses can be achieved. FS polymer coatings have shown high durability compared to paints [21] and are considered to have higher structural durability than thin SLIPS combined with a thin membrane and infused oil [20].

### 3.1. Coating Structures

**Figure 4:**
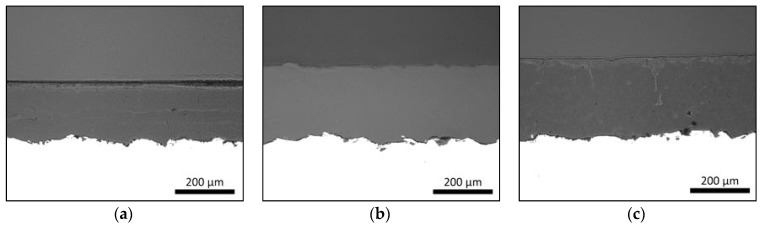
Flame-sprayed polymer (**a**) PE, (**b**) polyether ether ketone (PEEK) and (**c**) polypropylene (PP) coatings (middle part) on steel substrates (white part). Cross-sections. OM images.

Flame-sprayed PE, PEEK and PP polymer coatings own dense coating structures as presented in Figure 4. These coating structures correspond to the schematic presentation of dense coating in Figure 3a. Process parameters and heat-input affect the coating formation, adhesion between coating and substrate as well as denseness or porosity level inside the coating [22]. If a dense coating structure is produced, the temperature cannot be too high in order to avoid the defects, e.g., gas bubbles, caused by high-temperature gas. On the other hand, if the temperature is too low, particles are not melting enough, and pores can be formed to the structure. These dense FS polymer coatings are good examples of the icephobic coatings followed by the first approach of the coating design.

**Figure 5:**
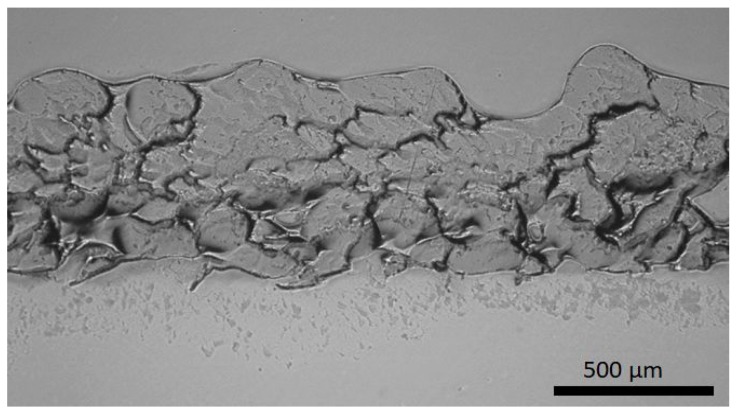
Structure of the FS porous polymer PE coating. Cross-section. OM image.

The second coating design approach relies on the SLIPS strategy. A porous coating structure was produced by flame spraying, and afterward, lubricant was impregnated to the structure. Flame-sprayed porous PE coating impregnated with silicone oil has shown excellent icephobicity [23]. Figure 5 shows the structure of the FS porous PE coating. Open and overall porosity can be detected in the structure, which is beneficial for lubricant impregnation.

### 3.2. Wettability of the Surfaces

**Figure 6:**
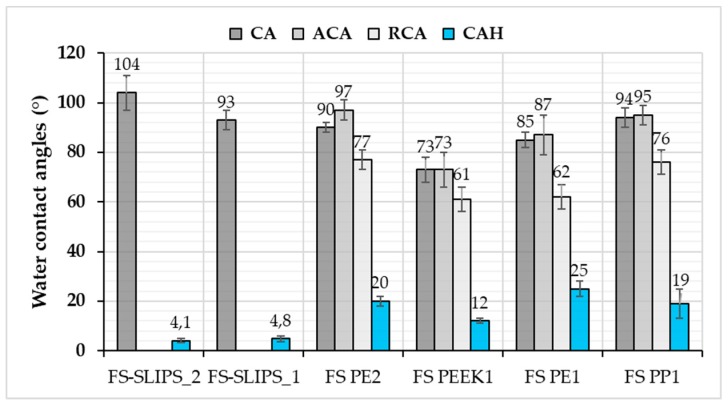
Water contact angles for flame-sprayed (FS) polymer coatings and FS-SLIPS. CA: static contact angle; ACA: advancing contact angle; RCA: receding contact angle; and CAH: contact angle hysteresis. Coatings are as-sprayed (1) or polished (2) prior testing.

In this study, FS PP and PE coatings, as well as FS-SLIPS, were hydrophobic or very close to that, Figure 6. This implies that the surfaces can resist the droplet from spreading. Generally speaking, hydrophobic and superhydrophobic surfaces have advantages in self-cleaning and anti-wetting purposes. Furthermore, in this study surface state after processing affected the wettability. Microstructures and surface topographies of the plasma sprayed coatings have shown to have an effect on wettability as presented, e.g., by Xu et al. [34] and Sharifi et al. [35]. Here, the degree of coating surface roughness can vary after flame spray processing, which can be smoothened by polishing as a post-treatment. Polished FS PE coating was hydrophobic (CA 90°) whereas as-sprayed coating was slightly hydrophilic (CA 85°). Surface roughness and uniformity influenced here by changing the wettability of the coatings. Based on this, FS coatings with certain surface quality have beneficial anti-wetting properties. Donadei et al. [22] have shown small differences in the wetting behavior between FS PE coatings sprayed with different spray parameters. Heat-input was varying between spray parameters and this influenced polymer degradation, surface roughness and thus, wettability. However, all FS PE coatings were hydrophobic in that study.

In addition to contact angle (CA) values, advancing (ACA) and receding (RCA) contact angle values, as well as contact angle hysteresis (CAH), indicate the wetting performance of the surfaces. CAH can be derived from the difference between ACA and RCA, demonstrating the overall droplet mobility. High CAH indicates differences between the ACA and RCA, which results from surface properties, such as local roughness variations, surface free energy, surface chemistry leading to altering droplet behavior. Flame-sprayed dense coatings have reasonable high contact angle hysteresis, but it can be reduced with FS-SLIPS surfaces. FS-SLIPS have low contact angle hysteresis and, therefore, water droplet mobility is high. This, in turn, is beneficial for slipperiness and the slippery properties of the surfaces.

Wettability of the different surfaces and coatings is presented in Table 2. In addition, ice adhesion values and surface roughness (Sa values) are collected to the table. In this study, different material and surface groups have been analyzed in order to get an understanding of the behavior and potential of FS polymer coatings for application areas, where icephobicity and/or non-wettability are the important properties, such as in energy, construction and building industries.

Roughness of the surface has influenced the wettability [37,38] together with other surface and material properties [39]. It can be beneficial at a certain level. For SLIPS surfaces, roughness, together with porosity, can help the oil to lubricate the structure and surface. On the other hand, if a dense coating is produced by using FS, smoothness plays a role in the surface properties of the coatings. Polished surfaces are smoother compared to the as-sprayed coatings. During the thermal spray process, roughness forms due to the nature of the particle adherence and coating formation from the particles and splats. Particle and splat sizes are affecting the roughness as well as post-heating of the surface. If the surface was strongly post-heated, it resulted as a smoother surface. Especially, this is the case with thermal spraying of polymer materials because they are heat-sensitive materials, having low melting points [3].

### 3.3. Ice Adhesion of the Surfaces

Ice adhesion of the surfaces can be measured with different techniques and this affects strongly the given values [36]. Therefore, it is important to describe ice accretion methods and parameters together with the results. In addition to this, ice adhesion measuring technique needs to explain, e.g., is it a centrifugal, pendulum or pushing/pulling type of testing. At the moment, there is not a clear way to compare the results measured with the different tests. However, the trend of the results can be seen and compared. In this study, all the tested surfaces were measured in the same way using the same ice accretion and ice adhesion test method. Therefore, the results can be directly compared. The centrifugal ice adhesion test (CAT) used in this study has been presented as a usable test method to screen different surfaces also by Laforte et al. [16].

**Figure 7:**
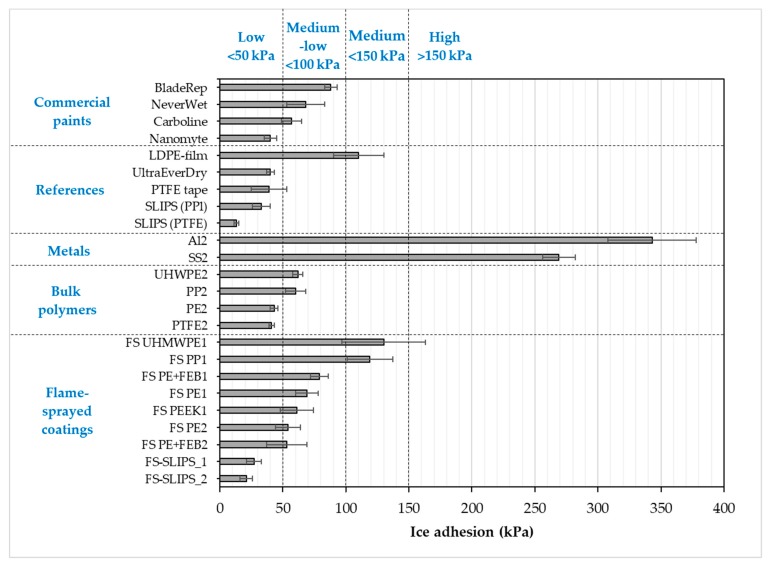
Ice adhesion strengths for several coatings and materials. Mixed-glaze ice accreted in IWiT and ice adhesion measured with CAT. One means as-sprayed and 2 polished surfaces. FS-SLIPS_1 contains a smoother porous structure and FS_SLIPS_2 a coarser porous structure.

**Figure 8:**
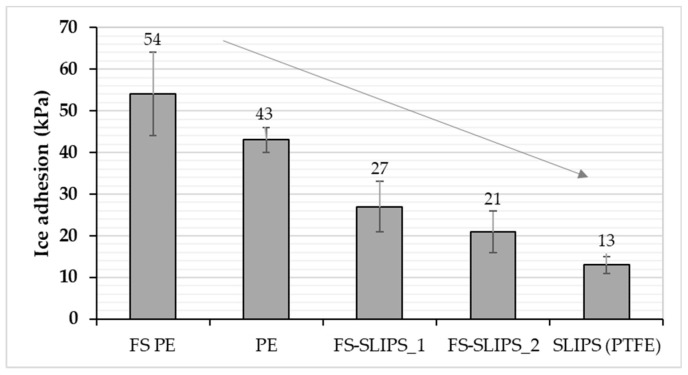
Comparison of ice adhesion between polished FS PE, polished bulk PE, FS-SLIPS and reference SLIPS (PTFE_memb + sil.oil_).

Ice adhesion is one of the indicators for icephobicity in such a way that low ice adhesion reflects good icephobicity and the ice is easily removed from the surface. If ice adhesion is high, then the ice is strongly adhered to the surface and icephobicity is low. We have used this definition for the ice adhesion strengths below 50 kPa, medium-low below 100 kPa, medium below 150 kPa and when high ice adhesion strengths are higher than 150 kPa. Ice adhesion is kept as extremely low when ice the value is below 10 kPa [20]. Figure 6 presents the ice adhesion strengths of tested surfaces. They were divided into different material groups in order to understand the behavior of different materials and surfaces. Metal surfaces had the highest ice adhesions due to their chemical properties whereas polymeric materials have generally relatively low ice adhesions due to their surface properties and slippery nature. From an application point of view, commercial paints were studied because they are used in the conditions where they can face the icing conditions, e.g., in wind turbine blades. The lowest ice adhesion strengths have been measured with SLIPS using porous membranes together with impregnated oil [20]. Thermally sprayed, here FS, polymer coatings showed medium-low ice adhesions. These are potential results taking account of the fact that durable coatings can be manufactured with thermal spray processing [21]. In addition, we combined durable FS PE coating and SLIPS concept and produced porous FS PE coating and impregnated oil to the porous structure [23]. This combination resulted in low ice adhesions as can be seen in Figure 7 and Figure 8. Flame-sprayed PE and PE-based composites, as well as PEEK coatings, had medium-low ice adhesions and FS UHMWPE and PP coatings and, in turn, medium ice adhesions. However, in flame spraying, coating properties can be influenced by process parameters [22] and further improvements are possible. The durability of the traditional SLIPS can be a challenge and, therefore, this novel way to produce SLIPS with high structural durability is shown to be the potential icephobic surface engineering solution. In addition to this, many icephobic surfaces rely on small scale, expensive or multistep methods, e.g., lithography [40], chemical synthesis [41] or sol-gel coatings [42], which can be avoided with thermal spraying. This, in turn, promotes thermal spraying to be a potential solution for smart and functional coating production [43], acting as environmentally friendly surface engineering solutions.

### 3.4. Comparison between Icephobicity and Surface Properties

**Figure 9:**
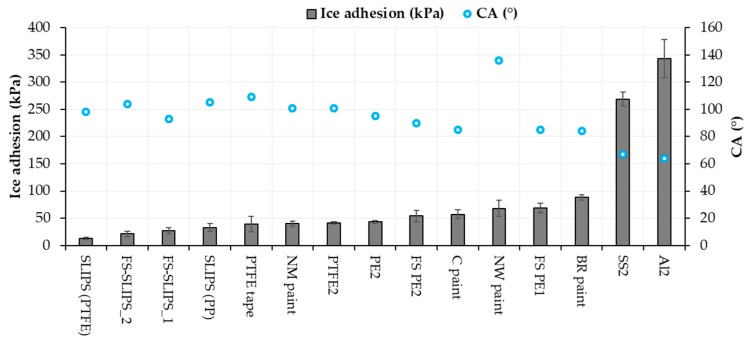
Ice adhesion and static contact angle relationships of the selected materials and surfaces.

Based on our previous studies [20,22,36] and the present results, the icephobicity, in general, does not have a correlation with high water contact angles in every surface technology. Icephobicity is affected by different aspects and clear explanations of the effect of different surface properties cannot be done. However, it was shown that the wettability and wetting properties of the surfaces had an influence on the ice adhesion. In many cases, hydrophobicity is beneficial for low ice adhesion. Superhydrophobic surfaces have shown their potential for icephobicity [18] but their environmental durability is a challenge [44]. In this study, surfaces, which had low or medium-low ice adhesions, were hydrophobic whereas metal surfaces with high ice adhesions were clearly hydrophilic, Figure 9 and Table 2. Similar findings have been reported by other research as well [45]. A comparison between ice adhesions and static water contact angles (CA) is drawn in Figure 9. 

**Figure 10:**
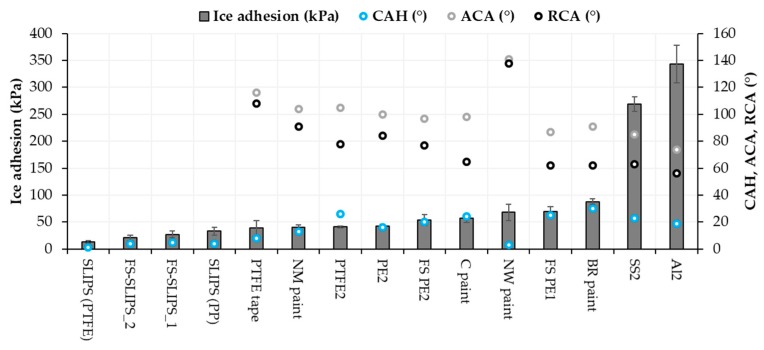
Ice adhesion and CAH, ACA and RCA relationships of selected materials and surfaces. CAH: contact angle hysteresis, ACA: advancing contact angle; and RCA: receding contact angle.

Contact angles (CA) showed only local wetting behavior of the surface and better indicator could be water contact angle hysteresis (CAH) measured with dynamic contact angle or sliding/roll-off contact angle measurements. This could indicate droplet mobility and can be related to icing as well with some materials and surfaces. The CAH of the surfaces is presented in Figure 10. For SLIPS, it has been calculated from roll-off angle measurements whereas for other surfaces in this study from dynamic contact angle measurements. Therefore, also advancing and receding contact angles for other surfaces than SLIPS are presented in Figure 10. As stated, the static contact angle does not clearly explain the ice adhesion, but a better relationship can be found by comparing contact angle hysteresis [46]. This was observed also here in the case of hydrophobic surfaces. SLIPS surfaces with low CAH had the lowest ice adhesions (Figure 10). 

**Figure 11:**
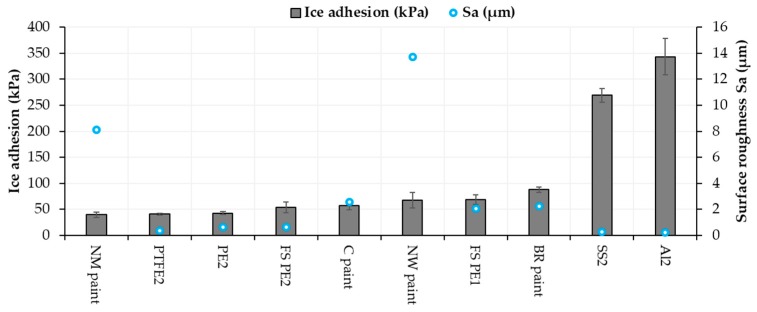
Ice adhesion and surface roughness (Sa) for selected materials and surfaces.

Roughness can influence ice adhesion although it is not a dominant factor itself. For example, mechanical interlocking as the adhesion mechanism between ice and surface can have an influence on the ice adhesion of textured surfaces [40]. Chemical properties, wettability and material properties play an important role, but it is difficult to separate their influences. Generally, higher roughness leads to surfaces with a larger number of crevices, which, in turn, gives more space for water droplets to stay. Therefore, it can increase ice adhesion. However, sometimes it is beneficial, for example, with the FS-SLIPS. FS-SLIPS_1 (fine) had lower surface roughness compared to FS-SLIPS_2 (coarse) without oil, and this resulted in lower ice adhesion for FS_SLIPS_2, when there was more area for oil to penetrate and stay on the surface. Some researchers have found that high roughness can be beneficial for lower ice adhesion [47], depending on the surface roughness pattern. Generally, it was reported that a smoother surface has lower ice adhesion [37,48,49]. This is also seen while comparing the same materials as here as-sprayed FS PE coatings and polished FS PE coatings. However, there are more dominant factors such as chemistry and surface energy and, therefore, metals (aluminum and stainless steel) had very high ice adhesion even though they are the smoothest surfaces. Figure 11 shows surface roughnesses and ice adhesions for selected surfaces. As a conclusion here, certain roughness is beneficial for low ice adhesion of the paints but with the FS polymer coatings and bulk polymers, a smoother surface gave lower ice adhesion. NW paint had high surface roughness compared to others and still reasonable ice adhesion. This could be explained by its high hydrophobicity and low CAH (Figure 10) [46].

## 4. Conclusions

Thermal spraying has shown its potential to produce multifunctional polymer coatings for icing conditions. Flame-sprayed polymer coatings had medium-low ice adhesions, indicating their good icephobicity. Furthermore, most FS coatings were hydrophobic, which, in turn, showed their potential for anti-wettability conditions. Structurally dense and well-adhered PE, PEEK and PP coatings were produced by using flame spraying. This is advantageous for environments where protection is needed. Flame spraying is the robust and fast coating manufacturing method, which is important in several industrial applications. Furthermore, the polymers used in this study are cheap materials, showing the potential of this processing-material combination for anti-icing and anti-wetting purposes.

Furthermore, SLIPS are interesting options as icephobic solutions. The lowest ice adhesion values were achieved with traditional SLIPS as well as with FS-SLIPS as a novel surface engineering solution by combining flame spraying of porous structure with impregnation of the oil. This way, we can have low ice adhesion surfaces as SLIPS, together with a durable structure, as thermally sprayed coatings. FS-SLIPS are shown their potential to act as slippery and icephobic surfaces.

Future work will focus on the widening of material selection for icephobic thermally sprayed coatings and testing of their durability under different environmental conditions with combining icephobicity. Laboratory scale icing testing is a good way to make the ranking of the surfaces, but application-related testing is needed for further development towards specific requirements in different conditions.

## Figures and Tables

**Table 1:** Coatings and surfaces used in this study divided into different material groups.

	Surface Treatment	Material	Form
**Flame-Sprayed Coatings**			
FS PE ^1^	^1^ As-sprayed	Polyethylene	Coating
FS PE ^2^	^2^ Polished	Polyethylene	Coating
FS PE+FEB ^1^	^1^ As-sprayed	Polyethylene + perfluoroethylene propylene	Coating
FS PE+FEB ^2^	^2^ Polished	Polyethylene + perfluoroethylene propylene	Coating
FS UHMWPE ^1^	^1^ As-sprayed	Ultra-high molecular weight polyethylene	Coating
FS PEEK ^1^	^1^ As-sprayed	Polyether ether ketone	Coating
FS PP ^1^	^1^ As-sprayed	Polypropylene	Coating
FS-SLIPS_1	SLIPS	Polyethylene (fine) + silicone oil (50 cSt)	SLIPS
FS-SLIPS_2	SLIPS	Polyethylene (coarse) + silicone oil (50 cSt)	SLIPS
**Bulk Polymers**			
PE ^2^	^2^ Polished	Polyethylene	Bulk plate
UHWPE ^2^	^2^ Polished	Ultra-high molecular weight polyethylene	Bulk plate
PP ^2^	^2^ Polished	Polypropylene	Bulk plate
PTFE ^2^	^2^ Polished	Polytetrafluoroethylene	Bulk plate
**Bulk Metals**			
Al ^2^	^2^ Polished	Aluminum	Bulk plate
SS ^2^	^2^ Polished	Stainless Steel	Bulk plate
**References**			
LDPE	As-received	Low-density polyethylene	Film
PTFE	As-received	Polytetrafluoroethylene	Tape
SLIPS (PTFE)	SLIPS	Polytetrafluoroethylene membrane (0.2 µm) + silicone oil (50 cSt)	SLIPS
SLIPS (PP)	SLIPS	Polypropylene membrane (0.2 µm) + silicone oil (50 cSt)	SLIPS
**Commercial Paints**			
BladeRep9	Painted, BR	Polyurethane	Paint
Carboline	Painted, C	Elastomeric	Paint
Nanomyte	Painted, NM	Nanocomposite	Paint
NeverWet	Painted, NW	Superhydrophobic	Paint
UltraEverDry	Sprayed	Superhydrophobic	Film

^1^ As-sprayed surface, ^2^ Polished surface

**Table 2:** Overview of the results divided by different material groups. Ice adhesion, contact angle (CA), advancing contact angle (ACA), receding contact angle (RCA), contact angle hysteresis (CAH) and surface roughness (Sa) of the materials and surfaces (± standard deviation).

Material or Surface	Ice Adhesion (kPa)	Contact Angle (°)	Adv. Contact Angle (°)	Rec. Contact Angle (°)	CA Hysteresis (°)	Roughness Sa (µm)
**Flame-Sprayed Coatings**						
FS PE ^1^	69 (±9) [21]	85 (±3)	87 (±8)	62 (±5)	25 (±3)	2.05 [21]
FS PE ^2^	54 (±10) [21]	90 (±2)	97 (±4)	77 (±4)	20 (±2)	0.64 [21]
FS PE + FEB ^1^	79 (±7) [21]	-	-	-	-	3.95
FS PE + FEB ^2^	53 (±16) [21]	75 (±5)	82 (±5)	65 (±)	18 (±0.1)	0.96 [21]
FS UHMWPE ^1^	130 (±33) [21]	91 [21]	-	-	-	1.65 [21]
FS PEEK ^1^	61 (±13)	73 (±5)	73 (±7)	61 (±5)	12 (±1)	-
FS PP ^1^	119 (±18)	94 (±4)	95 (±4)	76 (±5)	19 (±6)	-
FS-SLIPS_1	27 (±6) [23]	93 (±4)	*	*	4.8 (±1) *	3.0 ** [23]
FS-SLIPS_2	21 (±5) [23]	104 (±7)	*	*	4.1 (±0.9) *	38.8 ** [23]
**Bulk Polymers**						
PE ^2^	43 (±3) [36]	95 (±2)	100 (±2)	84 (±2)	16 (±0.6)	0.64
UHWPE ^2^	62 (±4) [36]	84 (±2)	-	-	-	-
PP ^2^	60 (±8) [36]	93 (±2)	-	-	-	-
PTFE ^2^	41 (±2) [36]	101 (±0.4)	105 (±4)	78 (±6)	26 (±2)	0.38
**Bulk Metals**						
Aluminum ^2^	343 (±35) [21]	64 (±2)	74 (±1)	56 (±3)	19 (±3)	0.26 [21]
Stainless steel ^2^	269 (±13) [21]	67 (±1)	85 (±1)	63 (±3)	23 (±4)	0.23 [21]
**References**						
LDPE-film	110 (±20) [28]					-
PTFE tape	39 (±14) [20]	109 (±3)	116 (±1)	108 (±3)	8 (±2)	-
SLIPS (PTFE_memb + sil.oil_)	13 (±2) [20]	98 (±2) [20]	*	*	1 (±0.5) * [20]	-
SLIPS (PP_memb + sil.oil_)	33 (±7) [20]	105 (±2) [20]	*	*	4 (±2) * [20]	-
**Commercial Paints/Films**						
Blade Rep9	88 (±5) [21]	84 (±2)	91 (±0.6)	62 (±0.5)	30 (±0.7)	2.24 [21]
Carboline	57 (±8) [36]	85 (±1)	89 (±3)	65 (±2)	24 (±0.4)	2.55
Nanomyte	40 (±5) [36]	101 (±1)	104 (±2)	91 (±6)	13 (±4)	8.1
NeverWet	68 (±15)	136 (±3)	141 (±1)	138 (±1)	3.3 (±0.1)	13.71
UltraEverDry	40 (±3) [29]	-	-	-	-	-

^1^ As-sprayed surface; ^2^ polished surface; * from roll-off angle measurements; ** Sa value of porous FS PE coating without oil; - not analyzed.
